# Metabolic Consequences of Chronic Alcohol Abuse in Non-Smokers: A Pilot Study

**DOI:** 10.1371/journal.pone.0129570

**Published:** 2015-06-23

**Authors:** Obiamaka Obianyo, Yan Liang, Ellen L. Burnham, Ashish Mehta, Youngja Park, Karan Uppal, Frank L. Harris, Dean P. Jones, Lou Ann S. Brown

**Affiliations:** 1 Division of Pulmonary, Allergy & Critical Care Medicine, Emory University School of Medicine, Atlanta, Georgia, United States of America; 2 Division of Pulmonary and Critical Care, Department of Medicine, University of Colorado at Denver and Health Sciences Center, Denver, Colorado, United States of America; 3 College of Pharmacy, Korea University, Sejong City, Korea; 4 Atlanta Veterans’ Affairs and Emory University Medical Centers, Decatur, Georgia, United States of America; 5 Division of Neonatal-Perinatal Medicine, Department of Pediatrics, Emory University School of Medicine, Atlanta, Georgia, United States of America; 6 Children's Healthcare of Atlanta Center for Developmental Lung Biology, Atlanta, Georgia, United States of America; Medical University of South Carolina, UNITED STATES

## Abstract

An alcohol use disorder (AUD) is associated with an increased susceptibility to respiratory infection and injury and, upon hospitalization, higher mortality rates. Studies in model systems show effects of alcohol on mitochondrial function, lipid metabolism and antioxidant systems. The present study applied high-resolution metabolomics to test for these changes in bronchoalveolar lavage fluid (BALF) of subjects with an AUD. Smokers were excluded to avoid confounding effects and compliance was verified by cotinine measurements. Statistically significant metabolic features, differentially expressed by control and AUD subjects, were identified by statistical and bioinformatic methods. The results show that fatty acid and acylcarnitine concentrations were increased in AUD subjects, consistent with perturbed mitochondrial and lipid metabolism. Decreased concentrations of methyl-donor compounds suggest altered one-carbon metabolism and oxidative stress. An accumulation of peptides suggests proteolytic activity, which could reflect altered epithelial barrier function. Two metabolites of possible microbial origin suggest subclinical bacterial infection. Furthermore, increased diacetylspermine suggests additional metabolic perturbations, which could contribute to dysregulated alveolar macrophage function and vulnerability to infection. Together, the results show an extended metabolic consequence of AUD in the bronchoalveolar space.

## Introduction

Alcohol abuse is a major worldwide health issue and is an important contributor to lung disease [1, 2]. Excessive alcohol consumption impairs the innate and adaptive immune responses, increasing the susceptibility to pulmonary infection and associated mortality [1, 3–5]. Ethanol metabolism also generates oxidative stress in the lung, which perturbs the alveolar epithelium and contributes to the etiology of acute respiratory distress syndrome (ARDS) and chronic obstructive pulmonary disease (COPD) [6, 7].

Attenuated immune response in the lung of alcohol use disorder (AUD) subjects is partially attributed to impaired phagocytic function, decreased GM-CSF receptor expression, decreased Nrf2 signaling, zinc deficiency, and altered redox state in the alveolar macrophages [2, 8, 9]. Additionally, excessive alcohol consumption disrupts epithelial barrier function, which increases the amount of protein found in the epithelial lining fluid [10]. Alcohol abuse also promotes mitochondrial dysfunction in both alveolar type II cells and alveolar macrophages and fatty acid oxidation is blocked due to inhibition of fatty acid-oxidizing dehydrogenases [8, 11, 12]. In the lung, alcohol-induced oxidative stress generates reactive oxygen species and decreases antioxidants with both intracellular and extracellular glutathione pools depleted in type II cells and alveolar macrophages [4]. Exogenous supplementation of zinc acetate, glutathione, or an antioxidant precursor, such as S-adenosylmethionine (SAM) or N-acetylcysteine, improved the phagocytic function of alveolar macrophages in cellular and animal models [9, 13–15].

Bronchoalveolar lavage fluid (BALF) is commonly analyzed in lung disorder studies as a way to sample the epithelial lining fluid and assess the metabolic composition of the alveolar space needed for the maintenance of immune cells and barrier function [16]. For instance, an NMR metabolomics analysis of human BALF demonstrated that amino acids and lactate are significantly enriched in the airways of children with Cystic Fibrosis (CF), consistent with reports of increased proteolysis and inflammation known to occur in the CF lung [17]. These findings were consistent with an independent metabolomics analysis of BALF collected from premature infants with respiratory distress syndrome and bronchopulmonary dysplasia, suggesting that similar inflammatory processes are occurring in both patient populations [18]. An LC-MS metabolomics analysis of BALF has also been used to identify metabolites differentially expressed in patients diagnosed with the Acute Respiratory Distress Syndrome (ARDS) [19]. When compared to controls, metabolomics analysis of BALF from otherwise healthy HIV-1 infected subjects identified increased pyochelin, a siderophore produced by *P*. *aeruginosa*, suggesting that *P*. *aeruginosa* may have been present in the lower airways of our otherwise healthy HIV-1 patients despite high CD4 counts and low viral loads [20].

In the present study, we performed a metabolomics analysis on BALF collected from subjects with and without an AUD diagnosis in an effort to identify dysregulated pulmonary metabolic processes. To avoid confounding effects of cigarette smoking, BALF from non-smokers were analyzed to observe the differential metabolites produced by alcohol abuse. The BALF of 10 AUD subjects and 10 controls were analyzed by dual-chromatography high-resolution mass spectrometry followed by statistical and bioinformatics analysis. Results show that alcohol abuse has extended metabolic consequences in the alveolar space including perturbations in fatty acid, amino acid, one-carbon, and polyamine metabolism.

## Methods

### Study Participants

Subjects with an AUD diagnosis were identified through the detoxification unit at the Veteran’s Affairs Hospital in Atlanta, GA. Controls were enrolled from those who replied to postings at the different Emory University hospitals as well as the community. The details of the recruitment process and selection criteria were previously reported [21]. Briefly, after informed consent was obtained, all subjects completed a pre-enrollment evaluation (visit 1) which included: 1) complete history and physical exam, 2) routine blood chemistries (basic chemistry, liver function tests, complete blood count, coagulation parameters), unless already performed as part of routine clinical care within the last 4 weeks, 3) urine pregnancy test (qualitative beta-HCG), 4) urine dipstick for cotinine, 5) spirometry (FEV1, FVC), 6) Short Michigan Alcohol Screening Test (SMAST) and Alcohol Use Disorders Identification Test (AUDIT) alcohol use questionnaires, and 7) BMI. The alcoholic status was confirmed by a score of > 3 on the SMAST survey. Subjects with a SMAST of 0 were considered as controls and underwent a similar screening procedure. For this study, only those control or alcoholic subjects without a smoking history were included. AUD subjects and controls were ineligible for the study if they met any of the following criteria: 1) prior medical history of liver disease (documented history of cirrhosis, total bilirubin ≥ 2.0 mg/dl, or serum albumin < 3.0), 2) prior medical history of gastrointestinal bleeding, 3) prior medical history of heart disease, 4) prior medical history of renal disease, 5) prior medical history of lung disease defined as an abnormal chest radiograph or spirometry, 6) concurrent illicit drug use defined as a toxicology screen for cocaine, opiates, or methamphetamines, 7) prior history of diabetes mellitus, 8) prior history of HIV, 9) failure of the patient to provide informed consent, 10) pregnancy, 11) age > 55, or 12) abnormal nutritional status. The nutritional status was assessed using the nutritional risk index with the subject’s albumin, current weight, and usual weight values in the following equation (17): NRI = 1.519 (albumin in g/l) + (current weight/usual weight) * 100 + 0.417. Subjects were considered to have a normal nutritional status if the NRI was ≥90.

### BALF Sample Collection

After informed consent was obtained, the lavage procedure on the non-smoking subjects with or without an AUD diagnosis was performed as previously described [21]. A flexible fiberoptic bronchoscope (Olympus Model BF-1T20D, Melville, NY) was passed transnasally into a subsegmental bronchus of the right middle lobe in all subjects. Once wedged, 150 ml of sterile saline (three 50-ml aliquots) were instilled and immediately aspirated into 50-ml suction traps under continuous low-pressure suction. The retrieved fluid was immediately filtered through coarse gauze and centrifuged (750 X g for 10 min) to remove cellular elements. The cell-free fluid (BALF) was aliquoted and stored at -80°C until future analysis.

### High Resolution Mass Spectrometry-based Metabolic Profiling of BALF

Samples were extracted and analyzed by liquid chromatography-high-resolution mass spectrometry (LC-FTMS) as previously described [20, 22–24]. Briefly, 100 μl aliquots of BALF were treated with an acetonitrile (2:1, v/v) solution containing an internal standard mix, centrifuged at 14,000 x g for 5 minutes at 4°C to remove protein, and then maintained at 4°C until injection. Data were collected by a Thermo LTQ-FT mass spectrometer (Thermo Fisher, San Diego, CA) for *m/z* 85 to 850 over 10 minutes with each sample analyzed in triplicate. Peak extraction and quantification of ion intensities were performed by an adaptive processing software package (apLCMS) designed for use with LC-FTMS data. Differentially expressed features were identified using in-house software package and MetaboAnalyst [25]. The in-house analysis utilized a log(2) transformation and normalization of the features, centering around the median, and LIMMA-based hypothesis testing with a false discovery rate (FDR) for significant features at *q* = 0.05. Resultant metabolome-wide association study (MWAS) data are displayed as Manhattan plots, where −log *p* for individual metabolites are plotted as a function of *m/z*. The predictive accuracy of the FDR features was evaluated by a 10-fold cross-validation using a Support Vector Machine. Two-way hierarchical clustering analysis of subjects and metabolites was performed using LIMMA [26]. MetaboAnalyst was used to perform partial least squares discriminant analysis (PLS-DA) after preprocessing the data with log transformation and autoscaling. Significant features were annotated using the online databases Metlin and Human Metabolome Database (HMDB) [27, 28]. The database searches were performed with a mass tolerance of 10 ppm, searching for commonly observed mass spectral adducts (i.e. M+H, M+Na, M+H-H_2_O).

### Measurement of Antioxidants and Oxidative Stress Markers

The BALF concentration of 8-iso-Prostaglandin F2α (F8-isoprostane), a stable by-product of lipid peroxides generated during oxidative stress, was quantitated by an ELISA assay (Cayman Chemical). The limit of detection was 2.7 pg/ml and the results are expressed relative to the BALF protein concentration or on a per mL basis. The cysteine and cystine concentrations of the BALF were determined by HPLC analysis after dansylation, as previously described [29]. Methionine and S-adenosylmethionine were also determined by HPLC analysis after derivatization with the AccQ.Fluor reagent (Waters Corporation; Milford, MA) [30].

## Results

### Subject characteristics

Ten non-smoking controls and ten non-smoking AUD subjects were enrolled (Table 1). The average age of subjects with an AUD diagnosis was 5 years older than that of controls. In addition, AUD subjects included a greater percentage of males, although this was not statistically significant (Table 1).

**Table 1:** Subject Demographics.

	*Nonsmoker Controls*	*Nonsmoker Alcohol Use Disorder*
**N**	10	10
**Male (%)**	2 (20)	8 (80)
**Age (SD)**	41 (4)	47 (5)^b^

P-value:

^b^ p = 0.01 (compared to nonsmoker control).

### Measurement of oxidative stress markers in BALF

**Fig 1:**
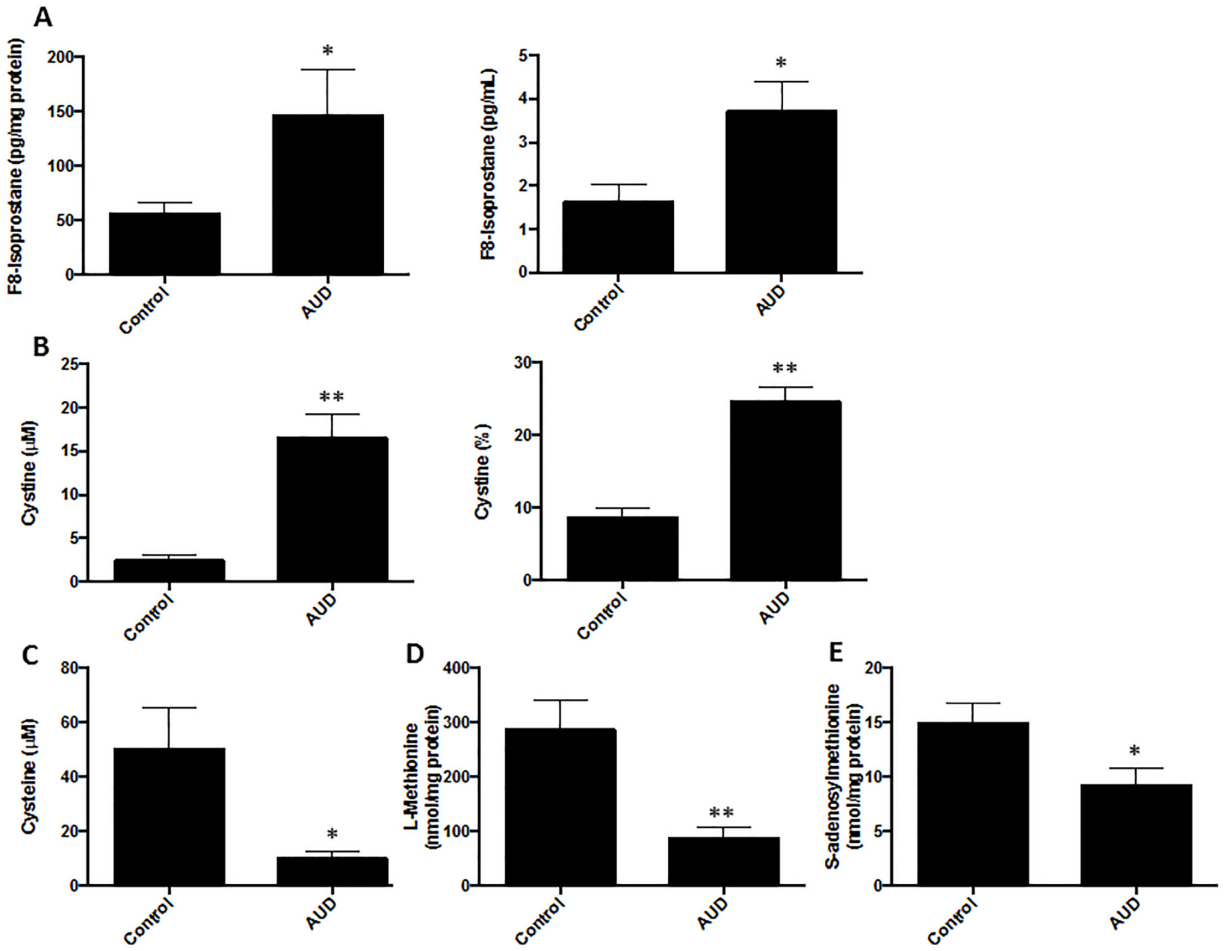
Oxidative stress markers are elevated in the BALF of AUD subjects. **A.** F8-Isoprostane in BALF was quantitated by an ELISA and expressed relative to the BALF protein concentration or on a per mL basis. **B.** Cystine was measured in the BALF by HPLC following dansylation and the concentration is expressed in μM or as a percentage of the total pool (cysteine + cysteine). **C.** After dansylation, cysteine was measured in the BALF by HPLC and the concentration expressed as μM. **D.** Methionine (nmol/mg BALF protein) was measured by HPLC following derivatization with the AccQ.Fluor reagent. **E.** S-adenosylmethionine (nmol/mg BALF protein) was measured by HPLC following derivatization with the AccQ.Fluor reagent.

Since alcohol abuse is known to exacerbate oxidative injury in the lung, the BALF was initially probed for selected markers of oxidation. F8-isoprostane, which is produced by the non-enzymatic oxidation of phospholipids, was significantly elevated in AUD subjects (Fig 1A). The concentration of cystine, the oxidized moiety of cysteine, was also markedly increased in the BALF from AUD subjects (Fig 1B), and the BALF concentrations of cysteine (Fig 1C) were correspondingly decreased. Decreased concentrations of methionine (Fig 1D) and S-adenosylmethionine (SAM) (Fig 1E), precursors for antioxidants and one-carbon metabolism, were also observed in AUD.

### Metabolomics analysis

**Fig 2:**
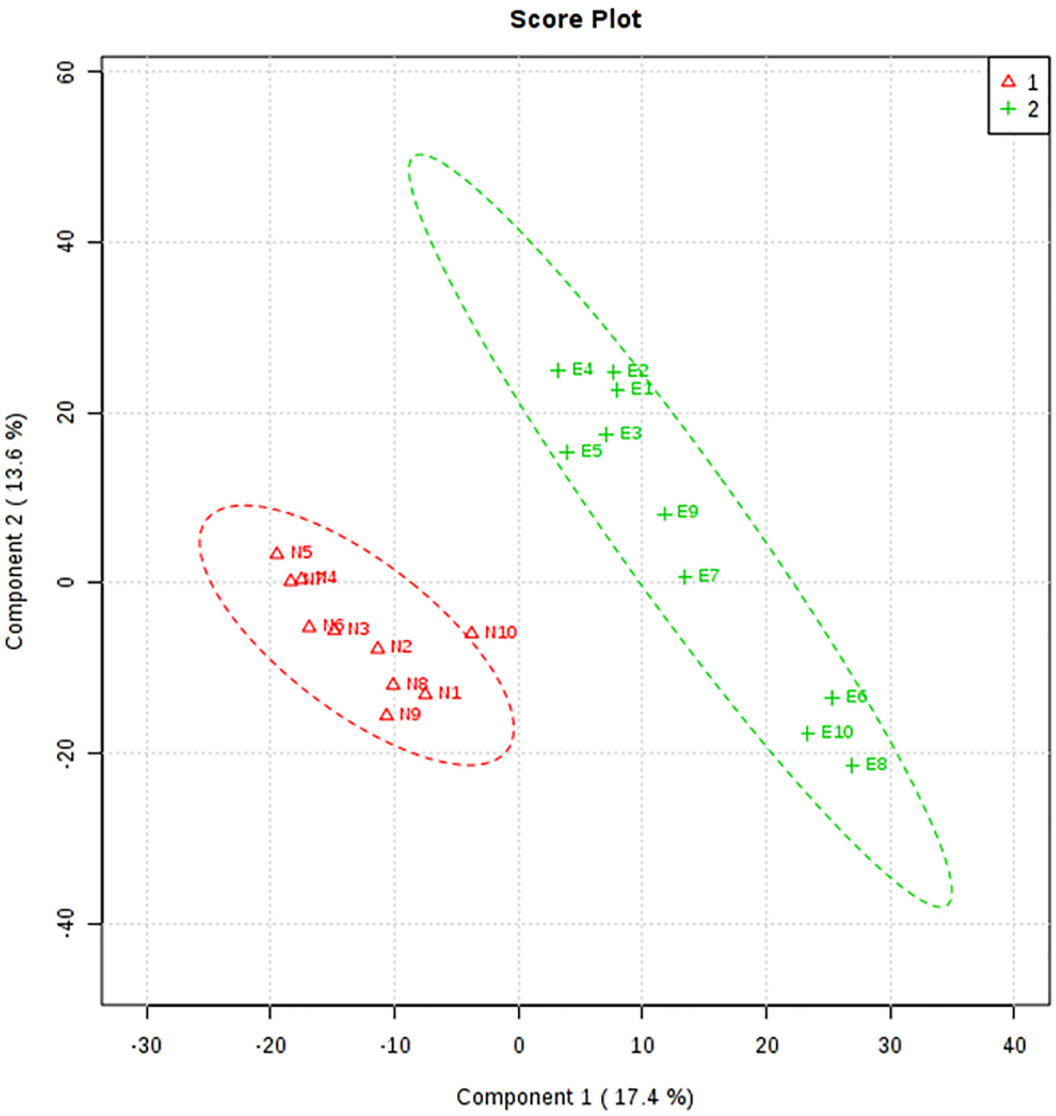
PLS-DA score plot of group separation along the two principal components. The results of the PLS-DA analysis separated the two groups by principal components 1 and 2, which are responsible for 30% of the variation. The q^2^ cross-validation was found to be 80%. N = no AUD diagnosis and “E” represents ethanol consumption, i.e. positive for an AUD diagnosis.

To identify additional metabolic effects of chronic alcohol abuse, the BALF samples were subjected to metabolomics analysis by LC-MS. After filtering data to remove metabolites that were present in less than 50% of the samples, data were analyzed based on the *m/z*, retention time, and ion intensities for 2688 features. Data containing the m/z and retention times from the metabolomics analysis are found in the supplement file entitled "S1 Table". To identify the features that were differentially expressed between controls and AUD subjects, we performed partial least squares-discriminant analysis, PLS-DA, which is a multivariate method that utilizes loading vectors to perform a correlation analysis in addition to group separation. Based on this analysis, the two groups were separated by the first two principal components (PC) (Fig 2). These PC accounted for 30% of the variation, and q^2^ demonstrated 80% cross-validation. The principal component 1 loadings were used to identify the top 10% of the features (160 *m/z* features) that contributed the most to the separation of the two groups.

**Fig 3:**
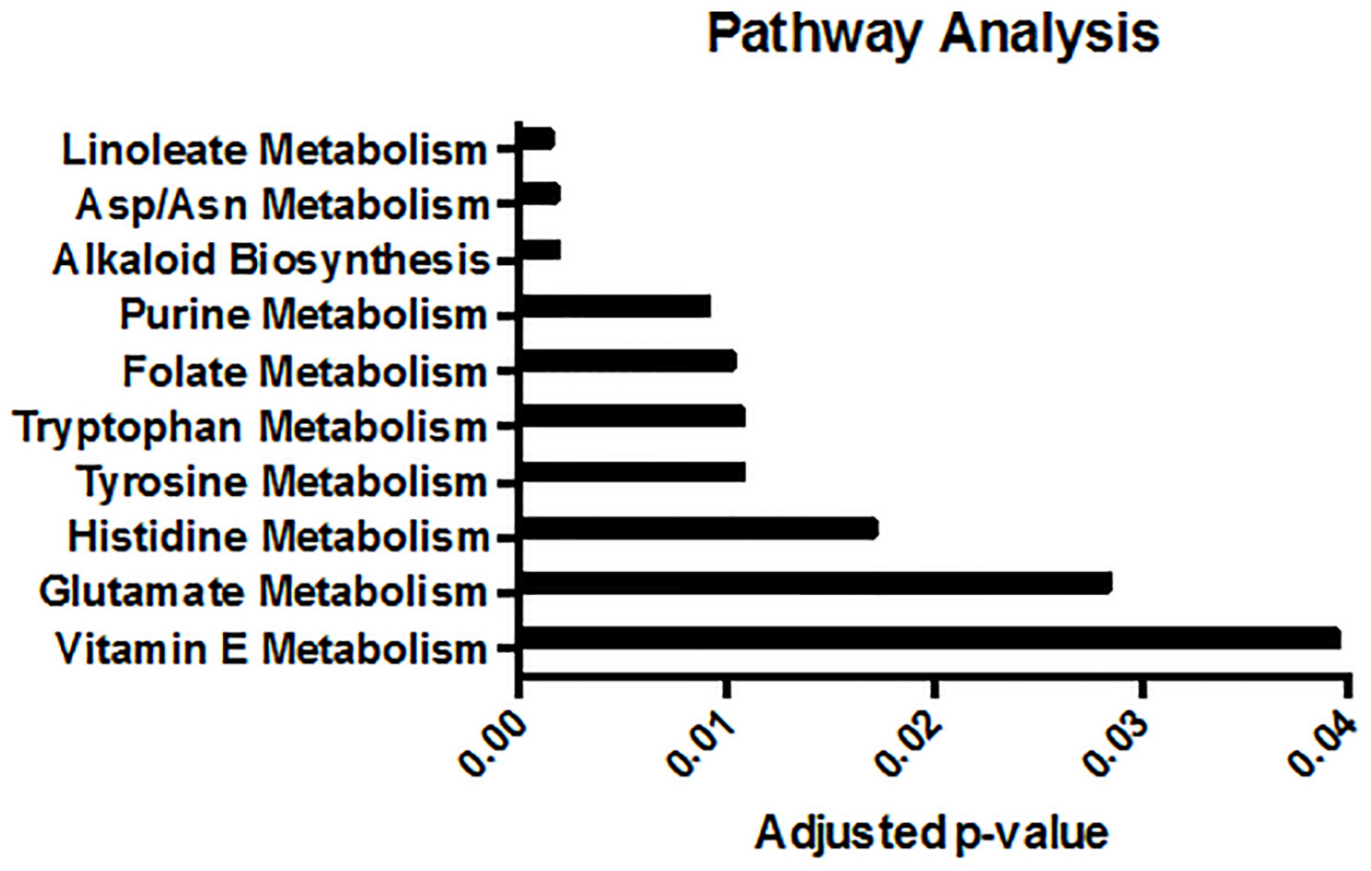
Significantly perturbed metabolic pathways in AUD subjects. Pathway analysis identified linoleate metabolism as the most significantly perturbed pathway in the BALF from AUD subjects.

The differentially expressed metabolites were then used to perform a pathway analysis to identify the metabolic pathways that are perturbed by an AUD. The top 10% of m/z features, identified by the PLS-DA analysis, were used for pathway mapping in the Mummichog software package [31]. The computational program matched the m/z features to metabolites and predicted the most active metabolic pathways and networks represented by the data. Linoleate metabolism was the most affected metabolic pathway in the BALF of AUD subjects (Fig 3). Folate metabolism and anti-inflammatory pathways seem to be significantly perturbed by alcohol abuse as well.

**Fig 4:**
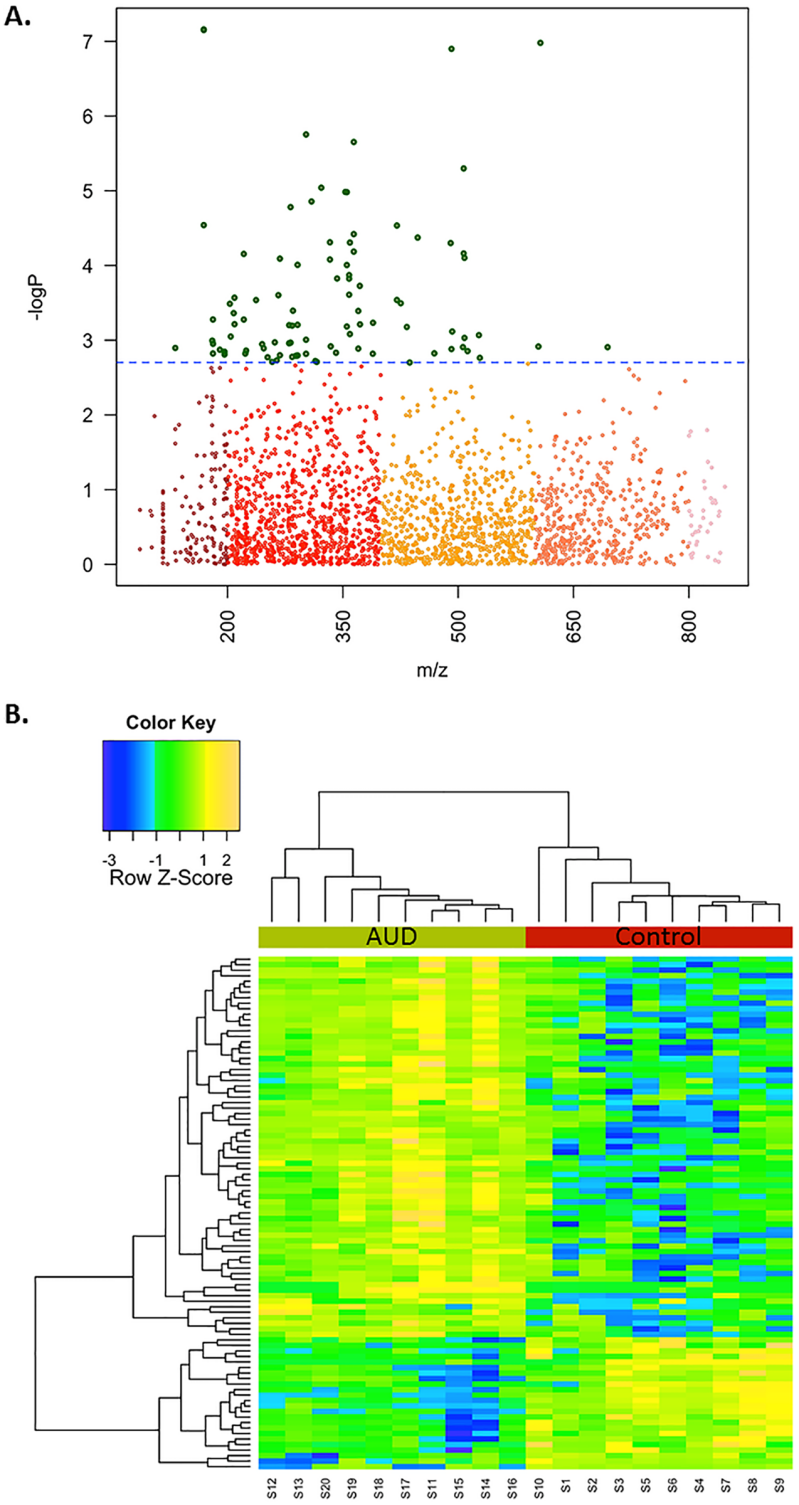
Differentially expressed features were identified by FDR analysis. FDR analysis was performed on the features identified in the BAL fluid of 10 alcoholic subjects and 10 controls. **A.** The Manhattan Plot demonstrates that 93 of the 2688 *m/z* features exceed the significance threshold (q = 0.05), denoted by the dashed line. **B.** Hierarchical clustering analysis is depicted in a heat map to demonstrate the separation of the two groups by differential expression patterns of 93 features.

As a complementary approach, we identified the significantly differentiated metabolites. To adjust for multiple comparisons, the false discovery rate procedure of Benjamini and Hochberg was used, resulting in 93 metabolites that were significantly different between the two groups at q = 0.05 (Fig 4A). Two-way hierarchical clustering analysis of these 93 metabolites showed that the individuals were distributed into two clusters, respectively including the controls and AUD patients. The 93 metabolites were distributed into 3 clusters, with 2 clusters increased in AUD patients and 1 cluster decreased in AUD patients (Fig 4B).

Of the 93 features, 49 features were also found to contribute to group separation by the PLS-DA analysis. Metlin and the Human Metabolome Databases (HMDB) were used to manually match these significant features to metabolite identities based on m/z values. In many cases, there were multiple potential identities; therefore, the most plausible identifications were assigned. Multiple fatty acids, including nitro-linoleic acid, were increased in AUD subjects, which is in accordance with the pathway analysis (Table 2). Similarly, the methionine and tryptophan derivatives are exemplary of altered folate and tryptophan metabolism (Tables 2 and 3). Other amino acids specified in the pathway analysis may be constituents of peptides, which may accumulate in the BALF of AUD subjects because of alcohol-induced proteolysis and altered epithelial barrier function. Manual annotation also identified novel perturbations such as bacterial and polyamine metabolic processes.

**Table 2:** Selected m/z features increased in AUD subjects.

	m/z	rt (min)	Annotation	Adduct
*Bacterial Metabolites*	341.1531	3.6	N-acetyl-8-O-methyl-neuraminic acid	M+NH_4_
	289.1028	4.6	N-formylmethionylphenylalanine	M+H-2H_2_O
*Oxidative Stress*	389.2501	9.0	10-nitrolinoleic acid	M+ACN+H
	370.0642	8.2	Pentenyl glucosinolate	M+H-H_2_O
*Polyamine Metabolites*	309.2264	9.3	N1,N12-diacetylspermine	M+Na
*Methionine Metabolites*	420.3175	3.3	N-palmitoyl methionine	M+CH_3_OH+H
	203.0393	8.7	1-propenyl-1-(propylsulfinyl)propyl disulfide	M+H-2H_2_O
*Tryptophan Metabolites*	291.0998	0.7	N-malonyltryptophan	M+H
*Fatty Acid Metabolites*	333.2017	0.8	Dihydroxypalmitic acid	M+2Na-H
	284.1493	0.6	3-hydroxyisovalerylcarnitine	M+Na
	355.1834	1.5	9-oxo-12,13-epoxy-10-octadecenoic acid	M+2Na+H
*Peptides*	490.2384	9.0	Peptide (N, N, R, S)	M+H
	302.1145	4.5	Peptide (E, F, G)	M+H-2H_2_O
	508.2053	4.1	Peptide (K, M, C, Y)	M+H-2H_2_O
*Miscellaneous*	237.1168	4.4	Octamethyltrisiloxane	M+H
	180.1380	0.7	3-oxopregn-4-ene-20beta-carboxaldehyde dioxime	M+H
	447.2919	9.0	2-fluoro-19-nor-22-oxa-1,25-dihydroxyvitamin D3	M+Na

**Table 3:** Selected m/z features decreased in AUD subjects.

	m/z	rt (min)	Annotation	Adduct
*Methionine Metabolites*	208.0390	0.6	S-methylmethionine	M+2Na-H
*Tryptophan Metabolites*	190.0496	0.8	Kynurenic acid	M+H
*Fatty Acid Metabolites*	280.1905	4.6	Phosphatidylserine	M+2H+Na
	169.1333	4.0	3-hydroxy-N6,N6,N6-trimethyl-L-lysine	M+H-2H_2_O
*Peptides*	694.3345	1.4	Peptide (W, W, V, Y)	M+ACN+H
*Miscellaneous*	245.0320	4.1	N6-O-disulfo-D-glucosamine	M+H-H_2_O
	204.1381	8.0	3-methyl-2-phenylbutanal	M+ACN+H

**Fig 5:**
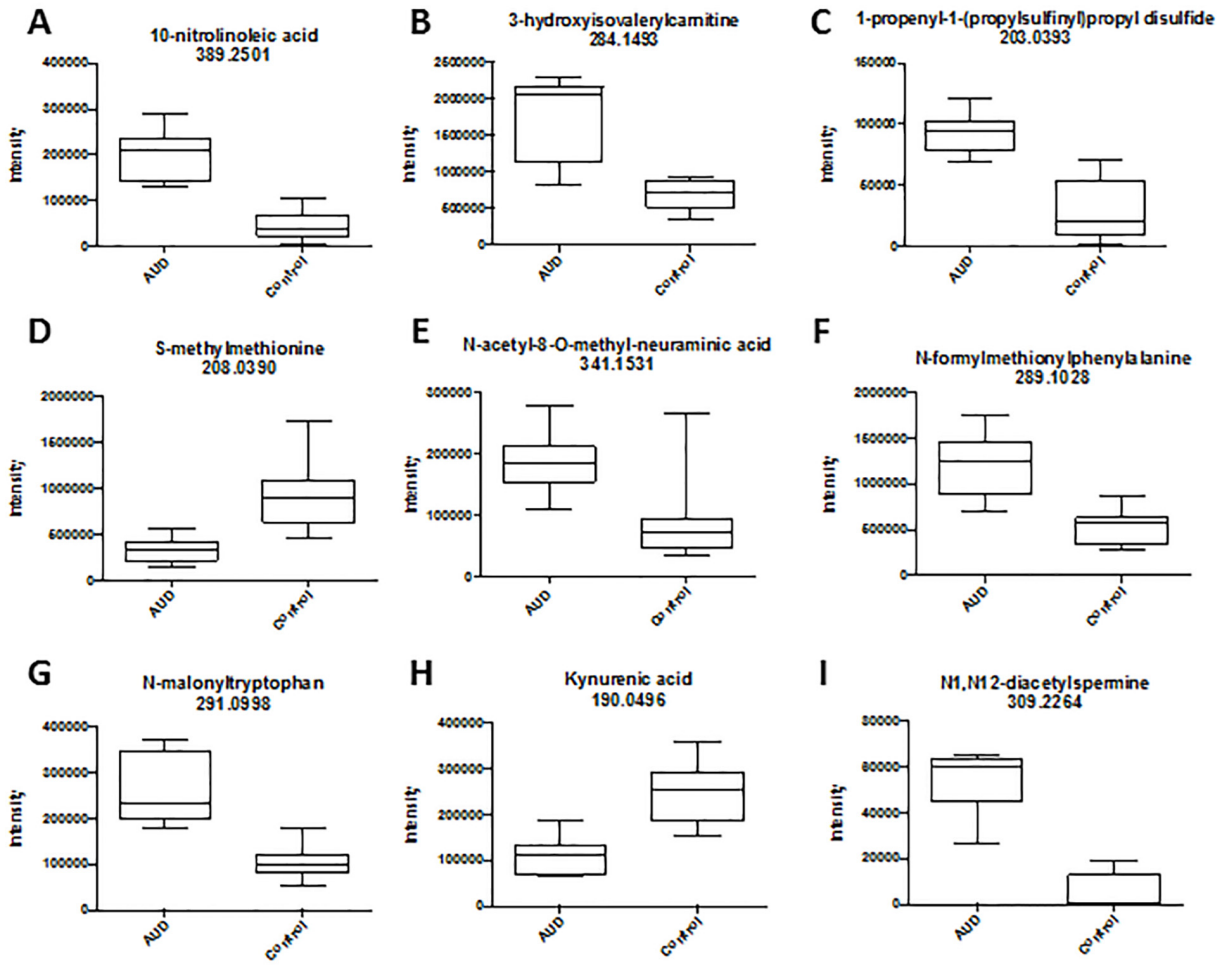
Box-and-whisker plots of selected significant features. The plots compare the mean intensity of each feature between the two groups.

As noted in the pathway analysis and manual annotation, linoleate metabolism was significantly altered. Specifically, the fatty acid 10-nitrolinoleic acid was significantly increased in the BALF of AUD subjects (Fig 5A). In addition, 3-hydroxyisovalerylcarnitine was also increased in the BALF of AUD subjects, in accordance with previous reports that alcohol abuse alters lipid metabolism (Fig 5B) [12]. As suggested by the decreased methionine and SAM in the BALF of AUD subjects (Fig 1), metabolomics analysis identified an increase in the methionine degradation product 1-propenyl-1-(propylsulfinyl)propyl disulfide (Fig 5C) as well as a decrease in SAM (Fig 5D). Bacterial products were also increased in the BALF of AUD subjects including N-Acetyl-8-O-methylneuraminic acid (Fig 5E), a bacterial cell wall component, and N-formylmethionylphenylalanine (fLMP), a bacterial chemotactic peptide (Fig 5F). Altered tryptophan metabolism was demonstrated by increased N-malonyltryptophan in the BALF of AUD subjects (Fig 5G) while kynurenic acid was depleted (Fig 5H). Furthermore, increased N1,N12-diacetylspermine in the BALF of AUD subjects suggests upregulated polyamine synthesis (Fig 5I).

## Discussion

Twenty million U.S. citizens suffer from an AUD [32], which is also associated with an increased risk of tuberculosis [33] and pneumonia [34–36], particularly serious Gram-negative or antibiotic-resistant strains of bacteria [36]. An AUD also increases intensive care use and the risk of ventilator-associated pneumonia [37–45] where it is associated with ~50% of all the ARDS cases with a 70% mortality rate [7, 21, 39, 40, 46]. One risk factor for ARDS is sepsis where both binge and chronic alcohol consumption have diverse and well-documented effects on the human immune system, leading to increased susceptibility to community acquired pneumonia and tuberculosis [34]. Despite the prevalence of alcohol dependence, treatment is rarely sought and tends to follow years of alcohol abuse, which causes deleterious effects on diverse lung functions [46]. Therefore, a detailed understanding of the metabolic differences between AUD and controls may provide new understanding of underlying factors by which alcohol abuse increases the risk of ARDS. Such understanding would facilitate the design of appropriate treatment strategies [9].

To address this question, the present metabolomics study was carried out and a diverse group of metabolites were found to differentiate the BALF of subjects with an AUD diagnosis from that of controls (Tables 2 and 3). Adducts of cotinine, a biomarker of cigarette smoke, were not observed in the BALF of either the control or AUD subjects further supporting that these subjects did not smoke. The cross-validation analysis indicated 80% correct classification; however, because of the small sample size or the manual annotation of the metabolites used for pathway analysis, this estimate may not be representative. In addition, we cannot rule the possibility that other medications, altered lung function, or other confounders may have contributed to altered metabolic pathways in the BALF of AUD subjects. Despite these considerations, the results of our study suggest that extended metabolic perturbations are associated with chronic alcohol abuse.

The predisposition of AUD subjects to lung infection and injury has been attributed to enhanced oxidative stress, decreased epithelial barrier function, mitochondrial dysfunction and impaired immune response. However, the underlying mechanisms as to how alcohol imposes these effects are unclear and there may be additional underlying metabolic perturbations that contribute to the increased risk of ARDS in the alcoholic lung. In our present study, the increased F8-isoprostane and cystine plus low cysteine, methionine and SAM levels in AUD subjects further supported oxidative stress and altered folate metabolism. In accordance with these findings, the methionine degradation product, 1-propenyl-1-(propylsulfinyl)propyl disulfide, was also elevated in AUD subjects. SAM arises via the methylation of methionine and is speculated to play a role in methionine storage and as a methyl donor. Furthermore, decreased SAM is also associated with alcohol abuse and aberrant methylation patterns of DNA and histones, important epigenetic mechanisms of transcriptional control [47–49]. In a rat model, dietary SAM (also known as vitamin U) restored SAM pools and attenuated alcohol-induced damage to the mucosa [50]. Similarly, we demonstrated in our adult rat model and our model of fetal alcohol exposure that dietary SAM restored alveolar epithelial cell function and decreased the risk of pneumonia [13, 51–55].

Pathway analysis and annotation identified linoleate metabolic pathway with the greatest number of altered metabolites, with 10-nitrolinoleic acid as one example. Another example of altered lipid metabolism was the increased 3-hydroxyisovalerylcarnitine. Carnitine plays an essential role in lipolysis, as it is responsible for transporting fatty acids from the cytosol to the mitochondria. In the presence of excessive amounts of acetyl-CoA, carnitine acyltransferase generates acylcarnitines, including the O-acylcarnitine, 3-hydroxyisovalerylcarnitine [56]. Alcohol consumption has also been associated with decreased plasma carnitine levels, and an increased acylcarnitine:free carnitine ratio [57]. Similarly, 3-hydroxyisovalerylcarnitine has been used as a biomarker for organic acidemias and fatty acid oxidation defects [58, 59].

The metabolomics analysis also suggest that the microbiome was affected by alcohol abuse. N-Acetyl-8-O-methylneuraminic acid, a bacterial cell wall component, and N-formylmethionylphenylalanine (fLMP), a bacterial chemotactic peptide, were elevated in AUD BALF. These observations suggest that there may be latent bacterial infections present in the AUD subjects. A similar observation was made in a metabolomics study of BALF from HIV-1-infected healthy individuals, suggesting a subclinical infection was present in the cohort [60]. Changes in the microbiome, or subclinical infection, may occur due to the immunosuppressed state of the alcohol-exposed alveolar space, which is unable to clear invasive bacteria. These results are consistent with an increased risk of infection but microbiome studies or other approaches are needed to determine if these products are indicative of a bacterial infection.

Spermine is a polyamine that is essential for biological processes including cellular proliferation and transcriptional and translational regulation [61]. The amino groups of the diamine can undergo acetylation, which is catalyzed by spermidine/spermine acetyltransferase (SSAT1) in the presence of acetyl-CoA. Upon acetylation, the diacetylspermine is excreted from the cell and has been observed in biological fluids and cell culture medium [62]. The increased diacetylspermine in the alcohol-exposed BALF suggests that alcohol promotes polyamine synthesis, acetylation, and excretion. Furthermore, this observation supports our previous studies demonstrating that chronic ethanol ingestion induces alternative activation of the alveolar macrophages, as demonstrated by increased expression of arginase 1 (Arg1) which shifts arginine away from nitric oxide synthase towards hydrolyzation to urea and ornithine, a precursor of polyamines like spermine [63].

In conclusion, this metabolomics study of BALF of subjects with an AUD demonstrated extended metabolic alterations in the lung. The results included increased fatty acid and acylcarnitine, consistent with perturbed mitochondrial and lipid metabolism; decreased methyl-donor compounds indicating altered one-carbon metabolism and oxidative stress; accumulated peptides suggesting increased proteolytic activity; increased microbial metabolites which could reflect subclinical infections; and increased diacetylspermine which could reflect increased polyamines by different cell types including alternatively activated alveolar macrophages. Additional studies are also needed to characterize the peptides identified to be differentially expressed between the two groups. Although the study was limited by the small sample size, widespread effects of alcohol abuse on BALF metabolite levels were observed with several validated by other methods. These results further suggest that high-resolution metabolomics may be a useful tool to identify previously unknown metabolic dysfunctions caused by chronic alcohol abuse and that lead to increased risks of respiratory infections or acute lung injury.

## Supporting Information

S1 Table Metabolomics Dataset. Data containing the m/z and retention times are presented in an Excel spreadsheet. Samples were ran in triplicate. Samples for the 10 nonsmoking AUD subjects are designated “E” and the 10 nonsmoking controls as “N”.
(XLSX)
